# Complaints against Hospitals

**Published:** 1898-08-27

**Authors:** 


### Complaints against Hospitals.

It is one of the ordinary features of what used to be called the "silly season" that it is fruitful in complaints as to the management of hospitals; complaints tacitly underlain by the assumption that, if either the members of the jury at a given inquest, or the sagacious author of an epistle from "A Constant Reader," had been in charge of the incriminated institution, a very much better state of things would have prevailed. It does not occur to the complainants, in a general way, that a hospital is a place supported by funds which are so far limited as to afford valid reasons for not admitting persons who seem likely to do well at home, or who, as often happens in the instances under consideration, would be very objectionable inmates of a ward. One of the commonest cases is that of a man who has received a blow on his head. Frequently he is the driver of some vehicle; is, if not actually drunk, at all events excited by alcohol, and, when taken to a hospital, is noisy and quarrelsome, declares there is nothing the matter with him, wishes to fight those who would detain him, and, if admitted, would be a source of serious and perhaps dangerous disturbance to other patients. He is allowed to go, the police taking him either to his home or to the station, and he lies down and appears to go to sleep. When attention is next called to him he is found to be comatose, and in a few hours he dies, without recovering consciousness. The ordinary explanation is that the blow has fissured the base of his skull, and, in doing so, has divided a very small blood vessel. The minute hæmorrhage does not at first produce any discoverable effect, unless, it may be, as one of the sources of the excitement; but as it continues it gradually produces sufficient compression of the brain to cause coma, and ultimately to kill. There is an inquest; the house-surgeon of the hospital, who acted to the best of his judgment, is solemnly commented upon by the jury, reproved by the coroner, and finally lectured by the house committee, may even be dismissed, unless his surgeon be a man of courage and common sense, who will place the whole matter in its true light before the lay authorities. We have known this history repeat itself on many occasions, and such conditions have been the subject of at least two sets of newspaper articles in the course of the last week.

The real question at issue is, of course, whether it be an advantage to a man to die in a hospital rather than in a police cell or in his own home. In such a case as that supposed, no one in the world could discover that bleeding was going on, so long as the patient was conscious; and no one, either then or at the time when the facts were rendered manifest by the occurrence of coma, could do anything to prevent or delay the inevitable result. Even if the hæmorrhage were on the surface of one of the hemispheres, the success of trephining would be extremely doubtful, although, of course, it might be attempted; but in the ordinary condition, when the fracture is at the base, it is quite certain that no such measure could be available. It would be well, as a rule, in any case of a noisy man who had fallen or had been struck, for the house surgeon to warn the police of the possibility of there being a serious injury by which insensibility might after a time be produced, and to direct that, in such an event, the patient should be immediately brought back. But neither the public nor jurymen should be permitted to believe that it would be proper to retain every noisy night-bird in a hospital, merely because here and there one may after a time pass into a condition of manifest danger. What is required is a much better organisation than exists at the present time for the medical supervision of persons who are in the charge of the police. If a person is sufficiently ill to be taken to hospital, he ought to receive some sort of medical attention when transferred to the police-station. Unfortunately, it would appear that refusal to admit a man to a hospital is too often taken as meaning that he is fit to be placed, unattended, in a cell at the police-station. So long, however, as a man is in charge of the police, and convicted of no crime, the police should make arrangements for his safe keeping; and the attempt to throw this responsibility on the hospitals is a gross abuse of charity.

